# Moderate Intense Physical Activity Depends on Selected Metabolic Equivalent of Task (MET) Cut-Off and Type of Data Analysis

**DOI:** 10.1371/journal.pone.0084365

**Published:** 2013-12-20

**Authors:** Hans van Remoortel, Carlos Augusto Camillo, Daniel Langer, Miek Hornikx, Heleen Demeyer, Chris Burtin, Marc Decramer, Rik Gosselink, Wim Janssens, Thierry Troosters

**Affiliations:** 1 Faculty of Kinesiology and Rehabilitation Sciences, Department of Rehabilitation Sciences, Katholieke Universiteit Leuven, Leuven, Belgium; 2 Respiratory Division and Rehabilitation, UZ Gasthuisberg, Leuven, Belgium; Pulmonary Research Institute at LungClinic Grosshansdorf, Germany

## Abstract

**Background:**

Accelerometry data are frequently analyzed without considering whether moderate-to-vigorous physical activities (MVPA) were performed in bouts of >10 minutes as defined in most physical activity guidelines. We aimed i) to quantify MVPA by using different commonly-applied physical activity guidelines, ii) to investigate the effect of bouts versus non-bouts analysis, and iii) to propose and validate a MVPA non-bouts cut-point to classify (in-) active subjects.

**Methods:**

Healthy subjects (n=110;62±6yrs) and patients with Chronic Obstructive Pulmonary Disease (COPD) (n=113;62±5yrs) wore an activity monitor for 7 days. Three Metabolic Equivalent of Task (MET) cut-offs and one individual target (50% VO_2_ reserve) were used to define MVPA. First, all minutes of MVPA were summed up (NON-BOUTS). Secondly, only minutes performed in bouts of >10 minutes continuous activity were counted (BOUTS). Receiver operating characteristic (ROC) curve analyses were used to propose and (cross-) validate new MVPA non-bout cut-points based on the criterion of 30 minutes MVPA per day (BOUTS). Likelihood ratios (sensitivity/[1-specificity]) were used to express the association between the proposed MVPA non-bout target and the MVPA bout target of 30 min*day^-1^.

**Results:**

MVPA was variable across physical activity guidelines with lowest values for age-specific cut-offs. Selecting a METs cut-point corresponding to 50% VO_2_ reserve revealed no differences in MVPA between groups. MVPA’s analyzed in BOUTS in healthy subjects were 2 to 4 fold lower than NON-BOUTS analyses and this was even 3 to 12 fold lower in COPD. The MVPA non-bouts cut-point of 80 min*day^-1^ using a 3 METs MVPA threshold delivered positive likelihood ratios of 5.1[1.5-19.6] (healthy subjects) and 2.3[1.6-3.3] (COPD).

**Conclusion:**

MVPA varies upon the selected physical activity guideline/targets and bouts versus non-bouts analysis. Accelerometry measured MVPA non-bouts target of 80 min*day^-1^, using a 3 METs MVPA threshold, is associated to the commonly-used MVPA bout target of 30 min*day^-1^.

## Introduction

The increasing burden of a physically inactive lifestyle has become a worldwide public health problem since it is directly related to morbidity and mortality and therefore does increase the health care costs [1]. For example, it is well established that physical inactivity is linked to a higher risk of developing type II diabetes [2], cancer [3], cardiovascular disease [4] and chronic obstructive pulmonary disease (COPD)[5]. Physical activity guidelines propose that the amount of time spent in moderate to vigorous physical activities (MVPA) to maintain health should be sufficiently high. The target of at least 30 minutes of MVPA per day, in bouts of at least 10 minutes, during at least 5 days of the week is proposed to distinguish active from inactive subjects [6,7]. A recent meta-analysis showed that 30 minutes of MVPA (bouts) per day during at least 5 days per week, as assessed by a self-completed questionnaire or an interview, was associated with a reduction in 19% of mortality risk [8]. Daily physical activity level (PAL; ratio of total energy expenditure over resting energy expenditure) is another important physical activity outcome which can be used to classify subjects as either active or inactive. It seems likely that the achievement of 1.7 PAL is needed to prevent the transition to overweight or obesity [9], one of the first and important consequences of a physically inactive lifestyle. The daily targets of ≥ 30 minutes of MVPA (bouts) and 1.7 PAL are commonly used and are embedded in two official physical activity recommendations by the World Health Organization [10,11]. 

The definition of MVPA is usually expressed in Metabolic Equivalents of Task (METs), but the absolute cut-points used vary between different guidelines. The current physical activity recommendation from the American College of Sports Medicine (ACSM) and the American Heart Association (AHA) (2007) defined moderate intense physical activity as ≥ 3 METs for all ages [6]. Because exercise capacity decreases with age, the ACSM/AHA recommendation for older adults can be prescribed at a relative MVPA cut-point, i.e. 50% of the subject’s oxygen uptake (VO_2_) reserve, in subjects older than 65 years or those with chronic diseases [7]. The ACSM Position Stand (2011) proposed cut-offs based on the age of people, rendering a threshold of 4.0 METs for people up to 65 years and 3.2 for subjects aged 65 years and older [12]. The current ACSM/AHA physical activity guidelines state that the 30 minute goal should be achieved in bouts of at least 10 minutes which complicates the assessment. The development of activity monitors makes accurate assessment (e.g. MVPA) of daily physical activity levels accessible. Since activity monitoring is increasingly being used and its data-analysis is frequently done without bouts, it would be worthwhile to propose a non-bout MVPA cut-point. 

Physical (in-) activity is also an important feature in chronic disease populations, such as patients with COPD. It is very well established that MVPA is significantly reduced in patients with COPD compared to a healthy control group [13]. A recent cohort study showed that a PAL > 1.7, as assessed by an activity monitor, was the strongest independent predictor of survival in patients with COPD [14]. Although PAL levels may have important predictive power, they cannot be easily translated into physical activity advice. Therefore, the impact of using different physical activity guidelines and data analysis ((non-)bouts) along with the validation of the “new” proposed MVPA non-bout cut-point is important in patient populations with chronic disease, such as COPD.

In a sample of healthy subjects and patients with COPD we aimed (i) to investigate to what extent the use of commonly-applied physical activity guidelines and targets on MVPA provide different results, (ii) to investigate the effect of bouts versus non-bouts analysis on MVPA (iii) to propose and (cross-) validate a non-bout MVPA cut-point equivalent to the commonly-used 30 minutes (bouts) criterion and to validate this proposed MVPA non-bout cut-point in healthy subjects and in patients with COPD.

## Materials and Methods

### Ethics Statement

The protocol was approved by the Medical Ethical Board of the University Hospitals Leuven (Leuven, Belgium), number B32220096387 and B322220095599. All subjects provided written informed consent. 

### Study participants

In this retrospective post-hoc cross-sectional study, subjects with COPD or without COPD ((never-)smokers) were included from two clinical trials (physical activity counseling study (NCT00948623) (i.e. adding a physical activity counseling program during pulmonary rehabilition in patients with moderate to very severe COPD, randomized controlled trial) and a case-control study of physical activity and comorbidities in mild to moderate COPD (NCT01314807) (patients with mild to moderate COPD and (never-)smokers without COPD) were included. The results of the physical activity levels and its association with clinical characteristics in the latter study have been published elsewhere [15]. Only baseline data from both trials were used in the present study. COPD was defined by post bronchodilator spirometry (ratio of forced expiratory volume in 1s (FEV_1_) over forced vital capacity (FEV_1_/FVC) < 0.70). The modified Medical Research Council (mMRC) dyspnea scale rates the type and magnitude of dyspnea according to five grades (from mMRC 0 to mMRC 4) of increasing severity [16]. Symptoms of dyspnea (mMRC scores) together with GOLD stages (spirometry) and/or exacerbation history were used to classify patients into the appropriate group (ranging from group A (low risk, less symptoms) to group D (high risk, more symptoms)) according to the revised GOLD classification [17]. . They were clinically stable and free of exacerbations for at least 4 weeks prior to the study. Subjects were excluded if they had orthopedic and/or musculoskeletal problems which would interfere with their movement patterns (e.g. arthritis).

### Study Procedures

All participants performed post-bronchodilator spirometry, physical exercise testing and assessment of daily physical activity. Spirometry was performed with standardized equipment and according to American Thoracic Society and European Respiratory Society guidelines [18]. FEV_1_ and FVC are given as absolute values and expressed as percentages of the predicted reference values [19]. Functional exercise capacity was determined by six minute walking distance (6MWD) [20]. Values were related to previously published reference values for the healthy Belgian population [21]. A symptom-limited incremental cycle ergometer test according to the ATS/ACCP statement on cardiopulmonary exercise testing [22], was used to assess the maximal exercise capacity (peak VO_2_) and the VO_2_ reserve (i.e. peak VO_2_ – resting VO_2_). The values of peak oxygen consumption were related to previously described reference values [23].

Subjects were instructed to wear a multisensor physical activity monitor (SenseWear Pro 2 Armband, Bodymedia, Pittsburgh, PA) for 7 consecutive days. Subjects from the physical activity counseling study were asked to wear the device during waking hours while subjects in the comorbidities study wore the activity monitor for 24 hours (except showering/bathing activities). This light weight (80 g) device is worn on the back of the upper right arm at the level of the triceps. It assesses accelerations in two planes using a bi-axial accelerometer. Furthermore skin temperature, near body temperature, heat flux and galvanic skin resistance are assessed and stored minute-by-minute for further analysis. Metabolic Equivalents of Task (METs) estimates from the SenseWear Pro 2 Armband were available through proprietary algorithms and equations by the manufacturer. METs data were downloaded in one minute bins for further analysis. For each minute of assessment, absolute or relative METs cut points were used to define MVPA, according to commonly-applied physical activity recommendations (see table **1**). METs values were converted to kilocalories to calculate total energy expenditure (TEE) estimates [24]. and were calculated as follows: TEE (kcal) = measured EE (with the SenseWear during wearing time) + predicted EE (based on resting energy expenditure (REE) during non-wearing time). Total energy expenditure estimates of this activity monitor have recently been validated against the gold-standard of doubly labeled water and indirect calorimetry in healthy adults [25] and in patients with COPD [26,27]. REE was calculated by gender-specific Harrison and Benedict prediction equations [28]. PAL was calculated by dividing daily TEE by resting energy expenditure. The subjects with activity monitoring during the whole day (24 hours); REE was calculated as the whole-night sleeping energy expenditure as measured with the SenseWear Pro 2 Armband [29]. PAL was calculated by dividing TEE by REE. Daily targets of ≥ 1.7 PAL and ≥ 30 minutes (bouts) of MVPA (≥ 3 METs) were used to classify subjects as physically active. A bout was defined as a period of at least 10 consecutive minutes above the recommended (MVPA) METs cut-point. A valid assessment was defined as a measurement of at least 5 days (weekend days + at least 3 weekdays) for at least 12 hours per day [30,31]. 

**Table 1:** Metabolic Equivalent of Tasks (METs) cut-points according to different physical activity recommendation.

**PA recommendation **	**METs cut-point to define (MVPA)**
ACSM/AHA	≥3.0 METs (for all ages)
ACSM/AHA_O_	≥3.0 METs (≤65 years) ≥50% VO_2_ reserve (METs) (>65 years)
50% VO_2_ reserve	Relative METs cut point corresponding to 50% VO_2_ reserve for each subject
ACSM 2011	≥4.0 METs (≤65 years) ≥3.2 METs (>65 years)

ACSM/AHA= current American College of Sports Medicine and American Heart Association recommendation (2007); ACSM/AHA_o_= American College of Sports Medicine recommendation for older adults (2007); 50% VO_2_ reserve= individual cut-point corresponding to 50% of oxygen uptake reserve; ACSM 2011: age-dependent cut-points from ACSM Position Stand 2011. MVPA= time spent in moderate to vigorous physical activities.

### Statistical analysis

Normal distribution was tested for all variables by a Shapiro-Wilk test [32]. Continuous variables were expressed as means with standard deviations (normal distribution) or as medians with interquartile ranges (skewed distribution). Categorical variables were expressed as proportions and testing between groups was done by a chi-square test. Comparisons between healthy subjects and patients with COPD were performed by either a parametric (unpaired t-test) or a non-parametric test (Wilcoxon-Mann-Whitney test). Comparisons in MVPA (skewed distribution) among different physical activity guidelines were tested by a Kruskal Wallis test (with Dunn’s post hoc testing). Linear regression analysis was performed to calculate MVPA (bouts) that corresponds to 1.7 PAL. The “split sample” approach was used to develop and cross-validate new MVPA non-bout cut-points [33]. We randomly divided our healthy population sample (n=110) in the 2:1 ratio and used the larger portion to develop new MVPA non-bout cut-points (calibration sample, n=73) and the remainder to cross-validate its performance (cross-validation sample, n=37). First, receiver operating characteristic (ROC) curve analyses were used to determine new MVPA non-bout cut-points based on the criterion of 30 minutes MVPA (bouts) (ACSM/AHA) (< 30 minutes MVPA per day; inactive, ≥ 30 minutes MVPA per day; active). ROC analyses identified the cut-points at which sensitivity and specificity were both maximized. Essentially, the challenge is to determine a cut-point that accurately captures “physical activity” (sensitivity) without capturing “inactivity” (specificity). The area under the ROC curve (AUC) is a measure of the accuracy of the cut-points. ROC AUC values of ≥ 0.90 are considered excellent, 0.80-0.89 good, 0.70-0.79 fair and <0.70 poor [34]. The new MVPA non-bout cut-points were subsequently confirmed using linear regression analysis. Secondly, ROC curve analyses were used to compare sensitivity and specificity for the new proposed MVPA non-bout cut-points against the criterion of 30 minutes MVPA (bouts) (ACSM/AHA), in both the healthy cross-validation sample (n=37) and in the sample of patients with COPD (n=113). Likelihood ratios (sensitivity/[1-specificity]) were calculated as a summarizing diagnostic accuracy statistic [35]. It expresses how many times more likely active subjects (≥ 30 minutes MVPA per day (BOUTS)) are to be classified as active according to the new MVPA non-bout target compared to inactive subjects (< 30 minutes MVPA per day (BOUTS)). A likelihood ratio greater than 1 indicates that the new MVPA non-bout target is associated with the standard activity target (≥ 30 minutes MVPA per day (BOUTS)). The further likelihood ratios are from 1 the stronger the evidence for the presence of an active lifestyle (based on the ≥ 30 minutes MVPA per day (BOUTS) target). Likelihood ratios above 10 are considered to provide strong evidence to rule in diagnoses in most circumstances. Data of the SenseWear Pro 2 Armband were downloaded with SenseWear Professional software 6.0. All statistical analyses were performed with SAS (version 9.3).

## Results

Two hundred fifty-three subjects were included in the present study; 110 subjects without COPD (comorbidities study) and 113 subjects with COPD (physical activity counseling study (n=55) and comorbidities study (n=58). Thirty subjects were excluded from the analysis (no valid physical activity assessment (n=23), musculoskeletal problems (n=2), cancer (n=2), rheumatoid arthritis (n=2), not motivated to participate (n=1). A general overview of the subjects’ characteristics (n=223) is provided in table **2**. Healthy subjects and patients with COPD did not differ statistically significant for age, sex and BMI. Lung function, exercise capacity (6MWD, W_max_ and peak VO_2_) and physical activity (steps and PAL) were significantly lower in patients with COPD (p<0.0001). A wide range of patients with COPD was recruited; FEV_1_ ranged from 0.6 L to 3.87 L, peak VO_2_ from 1.7 METs to 12.3 METs. The majority of patients with COPD were classified as quadrant A (50%) or quadrant D (42%).

**Table 2:** Overview of the subject’s characteristics.

Variable	Healthy subjects (n=110)	Patients with COPD (n=113)
Age	62 ± 6	62 ± 5
Sex (%men)	65	75
BMI	26.2 ± 3.9	26.1 ± 5.4
(Ex-)smokers, n (%))	52 (47)	58 (53)
FEV_1_ (L)	3.20 ± 0.76	1.91 ± 0.87*
FEV_1_ (%pred)	111 ± 18	65 ± 27*
FVC (L)	4.13 ± 0.94	3.62 ± 0.98*
FVC (%pred)	115 ± 17	100 ± 22*
FEV_1_/FVC (%)	77 ± 4	51 ± 14*
mMRC score 0/1/2/3/4 (n)	77/33/0/0/0	30/34/35/7/7*
Group A/B/C/D (n)	N/A	56/0/9/48
6MWD (m)	642 ± 82	516 ± 133*
6MWD (%pred)	97 ± 11	78 ± 19*
W_max_ (W)	173 ± 50	109 ± 55*
W_max_ (%pred)	117 ± 36	70 ± 32*
METs_max_	8.30 ± 2.26	6.01 ± 2.2*
Steps (per day)	9950 [7235-11918]	5725 [3002-8352]*
PAL (TEE*REE^-1^)	1.64 [1.52-1.76]	1.50 [1.28-1.71]*
Wearing time (hours*day^-1^)	23.08 ± 1.35	16.87 ± 4.48*

Data are expressed as means ± standard deviation or medians [interquartile range]. BMI= Body Mass Index; FEV_1_= Forced expired volume in the first second; FVC= Forced Vital Capacity; mMRC= modified Medical Research Council; 6MWD= six-minute walking distance; W_max_= Maximal workload; METs_max_= Maximal Metabolic Equivalent of Tasks; PAL= Physical Activity Level; TEE= Total Energy Expenditure; REE= Resting Energy Expenditure; N/A= not applicable *p<0.05 COPD versus healthy subjects.

**Figure 1:**
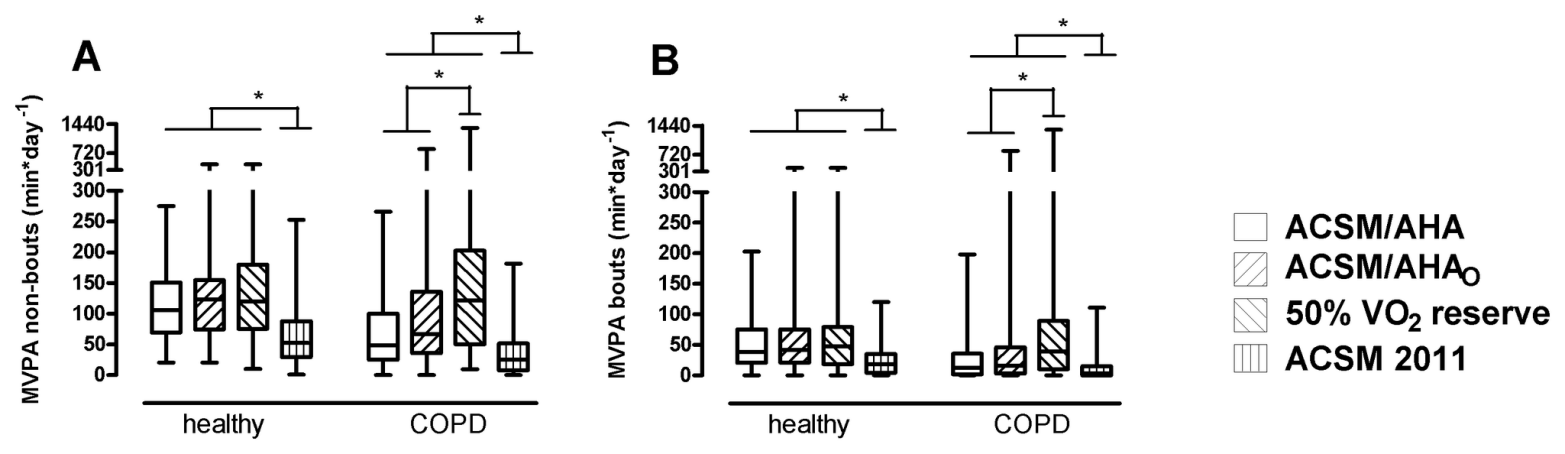
MVPA without (fig.1A) and with bouts (fig.1B) of at least 10 minutes according to different physical activity guidelines. ACSM/AHA= current American College of Sports Medicine and American Heart Association recommendation; ACSM/AHA_o_= American College of Sports Medicine recommendation for older adults; 50% VO_2_ reserve= individual cut-point corresponding to 50% of oxygen uptake reserve; ACSM 2011: age-dependent cut-points from the ACSM Position Stand 2011.

Analyzing MVPA with or without bouts of at least 10 minutes, revealed generally lower activity levels in patients with COPD compared to healthy controls (p<0.001, table **3**). No differences in MVPA were found between COPD and healthy subjects when using a relative cut-point of 50% VO_2_ reserve (table **3**). Using ACSM 2011 cut points resulted in significantly less minutes in MVPA in both healthy subjects and in patients with COPD (p<0.001, Figure **1A and 1B**). MVPA was significantly lower in ACSM/AHA_(O)_ guidelines compared to 50% VO_2_ reserve in patients with COPD (p<0.001, Figure **1A and 1B**) but not in healthy subjects. The median time spent in MVPA was 2 to 4 fold lower in healthy subjects when analyzing in bouts compared to non-bouts in all classifications (table **3**). This reduction in MVPA when using bouts was even more pronounced in patients with COPD (3 to 12 fold lower), especially for the ACSM 2011 cut points (table **3**). 

**Table 3:** Daily time spent in moderate to vigorous intense physical activities (MVPA) in healthy adults and patients with COPD, analyzed with and without bouts of at least 10 minutes.

PA recommendation	MVPA without bouts (min*day^-1^)		MVPA bouts (min*day^-1^)	
	Healthy (n=110)	COPD (n=113)	Healthy (n=110)	COPD (n=113)
ACSM/AHA	106 [70-151]	49 [26 - 99]*	39 [21-75]	13 [2 - 35]*
ACSM/AHA_O_	123 [74-155]	68 [36 - 135]*	42 [21-75]	16 [3 - 46]*
50% VO_2_ reserve	120 [76-180]	122 [51 - 202]	47 [19-79]	40 [11 - 87]
ACSM 2011	52 [30-88]	25 [8-52]*	18 [4-35]	3 [0-15]*

All data are expressed as medians [interquartile range]. PA; physical activity, ACSM/AHA= current American College of Sports Medicine and American Heart Association recommendation (2007) [7]; ACSM/AHA_o_= American College of Sports Medicine recommendation for older adults (2007) [6]; 50% VO_2_ reserve = individual cut-point corresponding to 50% of oxygen uptake reserve; ACSM 2011: age-dependent cut-points from ACSM Position Stand 2011 [12]. *p<0.001 COPD versus healthy subjects.

A minority of healthy subjects and patients with COPD were classified as physically active according to the 1.7 PAL target (37% and 26%, respectively). In the healthy subjects, daily PAL of 1.7 corresponds to 57 minutes MVPA per day(95% CI, 0 to 135 minutes) and 122 minutes MVPA per day (95% CI, 18 to 227 minutes), bouts and non-bouts respectively, with ACSM/AHA. 

Table **4** presents how many minutes in non-bouts are required to meet the 30 minutes MVPA bouts criteria for several cut-points in the calibration-sample. As the AUC was highest for the ACSM/AHA cut-point (80 minutes MVPA per day using a 3 METs MVPA threshold), this was taken forward for (cross-) validation in healthy subjects and in patients with COPD. Linear regression analysis confirmed the ROC analysis regarding the 80 minutes MVPA per day cut-point (30 minutes MVPA bouts is equivalent to 87 minutes MVPA per day (95% CI, 71 to 103 minutes MVPA per day)). 

**Table 4:** Development of new MVPA non-bouts cut-points in the healthy calibration sample (n=73).

**30 minutes MVPA (bouts) per day**	MVPA non-bout cut points (min*day^-1^)	Sensitivity % [95% CI]	Specificity % [95 % CI]	AUC
ACSM/AHA	80	83	85	0.89
		(58 to 96)	(73 to 93)	(0.81 to 0.98)
ACSM/AHA_O_	93	78	40	0.54
		(56 to 92)	(26 to 55)	(0.40 to 0.68)
50% VO_2_ reserve	75	85	30	0.56
		(65 to 96)	(17 to 45)	(0.42 to 0.70)
ACSM 2011	66	88	56	0.61
		(76 to 95)	(26 to 73)	(0.45 to 0.76)

ACSM/AHA= current American College of Sports Medicine and American Heart Association recommendation (2007) [7]; ACSM/AHA_o_= American College of Sports Medicine recommendation for older adults (2007) [6]; 50% VO_2_ reserve= individual cut-point corresponding to 50% of oxygen uptake reserve; ACSM 2011: age-dependent cut-points from ACSM Position Stand 2011 [12]. MVPA= time spent in moderate to vigorous physical activities.

### Validation of the proposed cut-points

**Figure 2:**
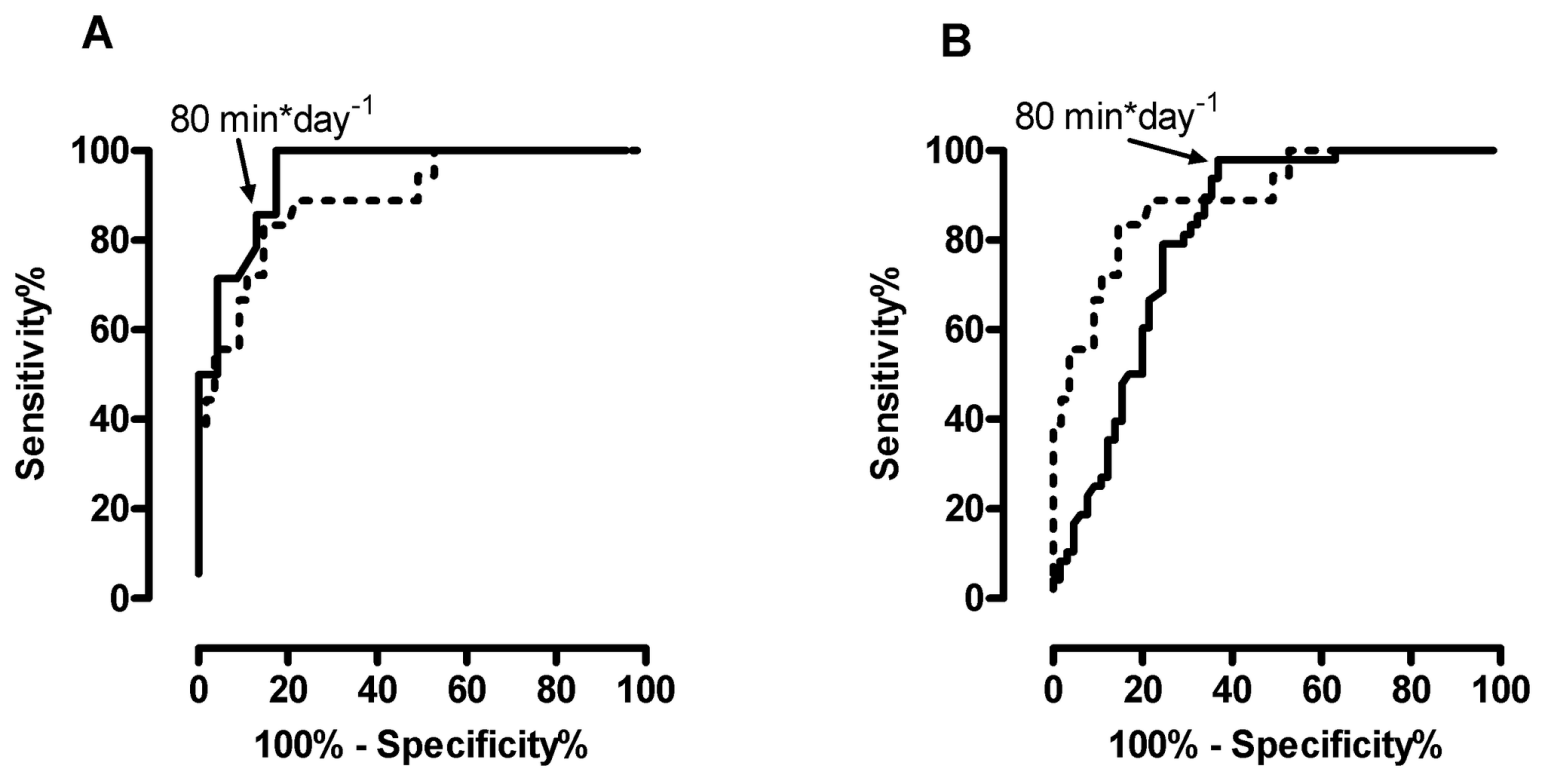
(Cross- ) validation of the new proposed non-bouts cut-point ROC curve showing the sensitivity and specificity percentages of the proposed MVPA non-bouts cut-point in the healthy cross-validation sample (n=37) (A) and in patients with COPD (n=113) (B). The dotted line represents the ROC curve of the healthy calibration sample.

Sensitivity and specificity percentages for this new MVPA non-bouts cut-point (80 min*day^-1^) in the healthy cross-validation sample of 37 subjects (age; 62±6 y, BMI; 25.8±4.1, 62% male, 51% >30 minutes MVPA per day (bouts)) (Figure **2A**) were 86% (57 to 98) and 83% (61 to 95), respectively. Finally, this proposed MVPA non-bout cut-point (80 min*day^-1^) was used to validate in a sample with COPD (n=113). Figure **2B** demonstrated the balance of sensitivity and specificity of this target in patients with COPD (98% sensitivity (95%CI, 89 to 100) and 58% specificity (95%CI, 45 to 70). Hence, the likelihood ratio (sensitivity/[1-specificity]) in the healthy subjects (cross-validation sample) and subjects with COPD was 5.1 [1.5-19.6] and 2.3 [1.6-3.3], respectively.

## Discussion

This study showed that (i) the time spent in moderate to vigorous physical activities (MVPA) was variable across different physical activity guidelines with lowest values for age-specific cut offs (ACSM 2011). Selecting a METs cut-point corresponding to 50% of VO_2_ reserve revealed no differences in MVPA between groups. A discrepancy was observed between 1.7 PAL and 30 minutes of MVPA as a guide to physical activity. A PAL of 1.7 corresponds to 57 minutes MVPA (BOUTS) and classifies the majority (69%) of subjects as inactive. Healthy subjects and particularly patients with COPD spend less time in MVPA when only bouts of 10 minutes were considered. (ii) The proposed MVPA non-bouts cut-point of 80 min*day^-1^, using a 3 METs MVPA threshold, was associated with the MVPA bouts cut-point of 30 min*day^-1^ in subjects with and without COPD. 

The reduction in MVPA by using the higher age-dependent METs cut-points of the ACSM Position Stand (2011) or by analyzing MVPA with bouts of at least 10 minutes can lead to confusion in assessing the physical activity status. Our data corroborate with those of Thompson et al. who concluded that in a middle-aged cohort (45-64y) 90% of the subjects could be variably described as either active or not sufficiently active, depending on the physical activity recommendation for MVPA that is applied [36]. In order to address this point, details of the selected METs cut-points and type of data analysis (bouts or non-bouts) should be provided in clinical trials, as advocated by the recent guidance of the ACSM [37]. Our data can help to convert non-bouts information to data with bouts of at least 10 minutes. The latter is becoming important since accelerometers, which enable non-bouts information, are increasingly used in assessing physical activity levels. Additionally, the definition of a bout should be clearly stated. We defined an MVPA bout as a consecutive period of at least 10 consecutive minutes above 3 METs. Two recent papers used a different ‘MVPA bouts definition’ by taking an accepted interruption period of 1 or 2 minutes below the threshold (3 METs) during a bout of at least 10 minutes [38] or by defining an bout as a consecutive period of at least 10 minutes above 3 METs according to their median intensity [39]. Due to these slight differences in ‘MVPA bouts definition’, it can be speculated that it would not have an important impact on the data interpretation, i.e. data-analysis in bouts will lead to significantly lower amount of MVPA compared to non-bouts analysis. However, the application of the MVPA bouts analysis should be further clarified in future research in order to set standardized recommendations for this type of data analysis [40].

To the best of our knowledge, this is the first study that used a METs cut-point corresponding to 50% of subjects’ individual VO_2_ reserve. No differences were found in MVPA between healthy subjects and COPD when this relative cut-point was applied for every subject. This result is counterintuitive and not in line with the general observation of inactivity in patients with COPD in the present data and others [41]. Patients with COPD have a reduced maximal exercise capacity and hence a low VO_2_ reserve compared to healthy controls. In subjects with severely reduced exercise capacity (e.g. COPD) 50% of VO_2_ reserve may represent a very light intense physical activity in absolute terms. This would lead to an overestimation in MVPA. Caution is warranted when 50% of VO_2_ reserve is applied to identify active patients in this population.

Another pitfall that needs consideration when classifying subjects as physically (in-) active is the required target that has been used. The daily target of 1.7 PAL and 30 minutes MVPA (bouts) with age-dependent METs cut-points (ACSM 2011) will classify the majority of subjects as physically inactive. The general aims behind the different physical activity recommendations are a possible explanation for this discrepancy. ACSM/AHA aims to improve and maintain the general health, where the ACSM position stand wants to develop and maintain the physical fitness. Thus, an important distinction has to be made between physical activities related to health (ACSM/AHA_(O)_) versus fitness (ACSM 2011). The cut-point of 1.7 PAL aims to prevent weight gain and has been previously reported to correspond to 45 to 60 minutes MVPA per day [9]. This is confirmed by our data, i.e. 57 minutes MVPA in bouts or 122 minutes MVPA in non-bouts are equivalent to 1.7 PAL. Therefore, 1.7 PAL is a very stringent target to classify active subjects compared to the 30 minutes MVPA (bouts) by ACSM/AHA_(O)_. A limitation of the present study was that part of the COPD patients (physical activity counseling study) did not have activity monitoring data for the recommended minimum of 22 hours per day to calculate PAL [42]. The wearing time of these patients was 16.87 ± 4.48 hours per day. Hence, we predicted the resting energy expenditure of these missing data (minutes) by the Harrison and Benedict equations [28]. Resting energy expenditure estimates of the SenseWear Pro 2 Armband and the Harrison and Benedict equations are successfully validated against indirect calorimetry [43,44]. Therefore, the average ~6 hours difference (sleeping time) with the subjects from the comorbidities study (23.08 ± 1.35 hours per day) probably would not have an important impact on the energy expenditure data.

The development and validation of a MVPA non-bouts cut-point has, to our knowledge, never been performed. This seemed useful since data analysis of MVPA in bouts of at least 10 minutes is not always performed by clinicians and researchers which lead to seemingly discrepant results. In a Swedish cohort of 1114 adults, 52% achieved the 30 minute goal when analyzed without bouts compared to only 1% when analyzed in bouts of at least 10 minutes [45]. The MVPA non-bouts cut-point of 80 min*day^-1^ delivered positive likelihood ratios of 5.1 [1.5-19.6] and 2.3 [1.6-3.3] in healthy subjects and subjects with COPD, respectively. This indicates that the proposed MVPA non-bout target of 80 min*day^-1^ is associated with the commonly-used activity target of MVPA bout target of 30 min*day^-1^. The stronger association in healthy subjects compared to subjects with COPD (as reflected by the higher likelihood ratio) can be explained by the lower specificity value in the subjects with COPD. The latter makes sense since our data showed that the reduction in MVPA when using bouts (compared to non-bouts) was more pronounced in patients with COPD (table 3). The more preserved daily MVPA when analyzed in non-bouts compared to bouts in COPD could be analyzed by the false positive rate (i.e. type I error or 1-specificity, which are the inactive subjects (<30 MVPA cut-point per day (bouts) who are labeled as active according to the 80 minute MVPA non-bout target). Indeed, the false positive rate was 42% compared to 17% in subjects with COPD compared to healthy subjects, respectively. To make stronger statement about this new MVPA non-bout target, future trials are needed to further validate whether long-term health benefits are obtained in subjects achieving these goals. Our study population consisted of healthy subjects and a specific chronic disease population (i.e. COPD). It might not be possible to generalize our results to other chronic disease populations. However the COPD subjects are representative in terms of physical activity levels when compared to other chronic diseases such as nefropathy [46], coronary heart disease [47] and metabolic syndrome [48]. It also has not be noted that the commonly-used recommendation of 30 minutes MVPA per day is based on the survey assessment of the leisure time physical activities by a physical activity questionnaire [49]. The objective assessment of all physical activity domains (household, occupational, leisure/recreation and active transport activities) by activity monitors (minute-by-minute data) may assume that this 30 minute recommendation would be higher when based on activity monitor data. The latter is already confirmed by the development and validation of the 80 minutes (non-bout) target. Finally, it remains unclear whether these MVPA targets are linked with adverse health-related outcomes, especially in the COPD population. This emphasizes the importance to validate the MVPA targets in future research. The present study can guide researchers and clinicians in collecting, analyzing and interpreting data of MVPA that are collected without bouts by accelerometers. This is worthwhile since the use of MVPA as a physical activity estimate as well as the transparency in collection and analysis of these data are strongly recommended [37].

In conclusion, the present study showed that MVPA varies depending on the selected PA guideline/targets and bouts versus non-bouts analysis. Accelerometry measured MVPA non-bouts target of 80 min*day^-1^, using a 3 METs MVPA threshold, is associated to the commonly-used MVPA bout target of 30 min*day^-1^.

## References

[1] Cadilhac DA, Cumming TB, Sheppard L, Pearce DC, Carter R et al. (2011) The economic benefits of reducing physical inactivity: an Australian example. Int J Behav Nutr Phys Act 8: 99. doi:10.1186/1479-5868-8-99. PubMed: 21943093. 21943093 PMC3192710

[2] Helmrich SP, Ragland DR, Paffenbarger RS Jr. (1994) Prevention of non-insulin-dependent diabetes mellitus with physical activity. Med Sci Sports Exerc 26: 824-830. PubMed: 7934754. 7934754

[3] Giovannucci E, Ascherio A, Rimm EB, Colditz GA, Stampfer MJ et al. (1995) Physical activity, obesity, and risk for colon cancer and adenoma in men. Ann Intern Med 122: 327-334. doi:10.7326/0003-4819-122-5-199503010-00002. PubMed: 7847643. 7847643

[4] Vatten LJ, Nilsen TI, Holmen J (2006) Combined effect of blood pressure and physical activity on cardiovascular mortality. J Hypertens 24: 1939-1946. doi:10.1097/01.hjh.0000244941.49793.f9. PubMed: 16957552. 16957552

[5] Garcia-Aymerich J, Lange P, Benet M, Schnohr P, Antó JM (2006) Regular physical activity reduces hospital admission and mortality in chronic obstructive pulmonary disease: a population based cohort study. Thorax 61: 772-778. doi:10.1136/thx.2006.060145. PubMed: 16738033. 16738033 PMC2117100

[6] Haskell WL, Lee IM, Pate RR, Powell KE, Blair SN et al. (2007) Physical activity and public health: updated recommendation for adults from the American College of Sports Medicine and the American Heart Association. Med Sci Sports Exerc 39: 1423-1434. doi:10.1249/mss.0b013e3180616b27. PubMed: 17762377. 17762377

[7] Nelson ME, Rejeski WJ, Blair SN, Duncan PW, Judge JO et al. (2007) Physical activity and public health in older adults: recommendation from the American College of Sports Medicine and the American Heart Association. Med Sci Sports Exerc 39: 1435-1445. doi:10.1249/mss.0b013e3180616aa2. PubMed: 17762378. 17762378

[8] Woodcock J, Franco OH, Orsini N, Roberts I (2011) Non-vigorous physical activity and all-cause mortality: systematic review and meta-analysis of cohort studies. Int J Epidemiol 40: 121-138. doi:10.1093/ije/dyq104. PubMed: 20630992. 20630992

[9] Saris WH, Blair SN, van Baak MA, Eaton SB, Davies PS, et al. (2003) How much physical activity is enough to prevent unhealthy weight gain? Outcome of the IASO 1st Stock Conference and consensus statement. Obes Rev 4: 101-114 10.1046/j.1467-789x.2003.00101.x 12760445

[10] FAO (1985) Energy and protein requirements. Report of a joint FAO/WHO/UNU Expert Consultation. 3937340

[11] WHO (2010) Global recommendations on physical activity for health. Geneva. 26180873

[12] Garber CE, Blissmer B, Deschenes MR, Franklin BA, Lamonte MJ et al. (2011) American College of Sports Medicine position stand. Quantity and quality of exercise for developing and maintaining cardiorespiratory, musculoskeletal, and neuromotor fitness in apparently healthy adults: guidance for prescribing exercise. Med Sci Sports Exerc 43: 1334-1359. doi:10.1249/MSS.0b013e318213fefb. PubMed: 21694556. 21694556

[13] Troosters T, Sciurba F, Battaglia S, Langer D, Valluri SR et al. (2010) Physical inactivity in patients with COPD, a controlled multi-center pilot-study. Respir Med 104: 1005-1011. PubMed: 20167463. 20167463 10.1016/j.rmed.2010.01.012 PMC3471783

[14] Waschki B, Kirsten A, Holz O, Müller KC, Meyer T et al. (2011) Physical activity is the strongest predictor of all-cause mortality in patients with COPD: a prospective cohort study. Chest 140: 331-342. doi:10.1378/chest.1119160. PubMed: 21273294. 21273294

[15] Van Remoortel H, Hornikx M, Demeyer H, Langer D, Burtin C et al. (2013) Daily physical activity in subjects with newly diagnosed COPD. Thorax 68: 962-963. doi:10.1136/thoraxjnl-2013-203534. PubMed: 23604460. 23604460 PMC3786635

[16] Bestall JC, Paul EA, Garrod R, Garnham R, Jones PW et al. (1999) Usefulness of the Medical Research Council (MRC) dyspnoea scale as a measure of disability in patients with chronic obstructive pulmonary disease. Thorax 54: 581-586. doi:10.1136/thx.54.7.581. PubMed: 10377201. 10377201 PMC1745516

[17] Vestbo J, Hurd SS, Agustí AG, Jones PW, Vogelmeier C et al. (2013) Global strategy for the diagnosis, management, and prevention of chronic obstructive pulmonary disease: GOLD executive summary. Am J Respir Crit Care Med 187: 347-365. doi:10.1164/rccm.201204-0596PP. PubMed: 22878278. 22878278

[18] Miller MR, Hankinson J, Brusasco V, Burgos F, Casaburi R et al. (2005) Standardisation of spirometry. Eur Respir J 26: 319-338. doi:10.1183/09031936.05.00034805. PubMed: 16055882. 16055882

[19] Quanjer PH, Stanojevic S, Cole TJ, Baur X, Hall GL et al. (2012) Multi-ethnic reference values for spirometry for the 3-95-yr age range: the global lung function 2012 equations. Eur Respir J 40: 1324-1343. doi:10.1183/09031936.00080312. PubMed: 22743675. 22743675 PMC3786581

[20] ATS Committee on Proficiency Standards for Clinical Pulmonary Function Laboratories (2002) ATS Statement: guidelines for the six-minute walk test Am J Respir Crit Care Med 166: 111-117 doi:10.1164/ajrccm.166.1.at1102. PubMed: 12091180.

[21] Troosters T, Gosselink R, Decramer M (1999) Six minute walking distance in healthy elderly subjects. Eur Respir J 14: 270-274. doi:10.1183/09031936.99.14227099. PubMed: 10515400. 10515400

[22] American Thoracic Society, American College of Chest Physicians (2003) ATS/ACCP Statement on cardiopulmonary exercise testing. Am J Respir Crit Care Med 167: 211-277. doi:10.1164/rccm.167.2.211. PubMed: 12524257. 12524257

[23] Jones NL, Makrides L, Hitchcock C, Chypchar T, McCartney N (1985) Normal standards for an incremental progressive cycle ergometer test. Am Rev Respir Dis 131: 700-708. PubMed: 3923878. 3923878 10.1164/arrd.1985.131.5.700

[24] McArdle WD, F.I. K., V.L. K., (2005) Energy Expenditure During Rest and Physical Activity, 3rd Edition. pp. 722-724.

[25] St-Onge M, Mignault D, Allison DB, Rabasa-Lhoret R (2007) Evaluation of a portable device to measure daily energy expenditure in free-living adults. Am J Clin Nutr 85: 742-749. PubMed: 17344495. 17344495 10.1093/ajcn/85.3.742

[26] Rabinovich RA, Louvaris Z, Raste Y, Langer D, Remoortel HV et al. (2013) Validity of physical activity monitors during daily life in patients with COPD. Eur Respir J, 42: 1205–15. PubMed: 23397303. 23397303 10.1183/09031936.00134312

[27] Van Remoortel H, Raste Y, Louvaris Z, Giavedoni S, Burtin C et al. (2012) Validity of six activity monitors in chronic obstructive pulmonary disease: a comparison with indirect calorimetry. PLOS ONE 7: e39198. doi:10.1371/journal.pone.0039198. PubMed: 22745715. 22745715 PMC3380044

[28] Harris JA, Benedict FG (1918) A Biometric Study of Human Basal. Metabolism - Proc Natl Acad Sci U S A 4: 370-373. doi:10.1073/pnas.4.12.370. 16576330 PMC1091498

[29] Watz H, Waschki B, Boehme C, Claussen M, Meyer T et al. (2008) Extrapulmonary effects of chronic obstructive pulmonary disease on physical activity: a cross-sectional study. Am J Respir Crit Care Med 177: 743-751. doi:10.1164/rccm.200707-1011OC. PubMed: 18048807. 18048807

[30] Miller GD, Jakicic JM, Rejeski WJ, Whit-Glover MC, Lang W et al. (2012) Effect of Varying Accelerometry Criteria on Physical Activity: The Look AHEAD Study. Obesity (Silver Spring), 20: 1122–6. PubMed: 22016090. 23505166 10.1038/oby.2012.118 PMC3430806

[31] Scheers T, Philippaerts R, Lefevre J (2012) Variability in physical activity patterns as measured by the SenseWear Armband: how many days are needed? Eur J Appl Physiol 112: 1653-1662. PubMed: 21874552. 21874552 10.1007/s00421-011-2131-9

[32] Razali NM, Wah YB (2011) Power comparisons of Shapiro-Wilk, Kolmogorov-Smirnov, Lilliefors and Anderson-Darling tests. Journal Statistical Modeling and Analytics 2: 21-33.

[33] Staudenmayer J, Zhu W, Catellier DJ (2012) Statistical considerations in the analysis of accelerometry-based activity monitor data. Med Sci Sports Exerc 44: S61-S67. doi:10.1249/MSS.0b013e3182399e0f. PubMed: 22157776. 22157776

[34] Metz CE (1978) Basic principles of ROC analysis. Semin Nucl Med 8: 283-298. doi:10.1016/S0001-2998(78)80014-2. PubMed: 112681. 112681

[35] Deeks JJ, Altman DG (2004) Diagnostic tests 4: likelihood ratios. BMJ 329: 168-169. doi:10.1136/bmj.329.7458.168. PubMed: 15258077. 15258077 PMC478236

[36] Thompson D, Batterham AM, Markovitch D, Dixon NC, Lund AJ et al. (2009) Confusion and conflict in assessing the physical activity status of middle-aged men. PLOS ONE 4: e4337. doi:10.1371/journal.pone.0004337. PubMed: 19183812. 19183812 PMC2629570

[37] Matthews CE, Hagströmer M, Pober DM, Bowles HR (2012) Best practices for using physical activity monitors in population-based research. Med Sci Sports Exerc 44: S68-S76. doi:10.1249/MSS.0b013e3182399e5b. PubMed: 22157777. 22157777 PMC3543867

[38] Scheers T, Philippaerts R, Lefevre J (2013) Compliance with different physical activity recommendations and its association with socio-demographic characteristics using an objective measure. BMC Public Health 13: 136. doi:10.1186/1471-2458-13-136. PubMed: 23409982. 23409982 PMC3599794

[39] Donaire-Gonzalez D, Gimeno-Santos E, Balcells E, Rodríguez DA, Farrero E et al. (2013) Physical activity in COPD patients: patterns and bouts. Eur Respir J 42: 993-1002. PubMed: 23258786. 23258786 10.1183/09031936.00101512

[40] Welk GJ, McClain J, Ainsworth BE (2012) Protocols for evaluating equivalency of accelerometry-based activity monitors. Med Sci Sports Exerc 44: S39-S49. doi:10.1249/MSS.0b013e3182399d8f. PubMed: 22157773. 22157773

[41] Vorrink SN, Kort HS, Troosters T, Lammers JW (2011) Level of daily physical activity in individuals with COPD compared with healthy controls. Respir Res 12: 33. doi:10.1186/1465-9921-12-33. PubMed: 21426563. 21426563 PMC3070642

[42] Watz H, Waschki B, Meyer T, Magnussen H (2009) Physical activity in patients with COPD. Eur Respir J 33: 262-272. PubMed: 19010994. 19010994 10.1183/09031936.00024608

[43] Kozey S, Lyden K, Staudenmayer J, Freedson P (2010) Errors in MET estimates of physical activities using 3.5 ml x kg(-1) x min(-1) as the baseline oxygen consumption. J Phys Act Health 7: 508-516. PubMed: 20683093. 20683093 10.1123/jpah.7.4.508

[44] Mackey DC, Manini TM, Schoeller DA, Koster A, Glynn NW et al. (2011) Validation of an armband to measure daily energy expenditure in older adults. J Gerontol A Biol Sci Med Sci 66: 1108-1113. PubMed: 21734231. 21734231 10.1093/gerona/glr101 PMC3172563

[45] Hagströmer M, Oja P, Sjöström M (2007) Physical activity and inactivity in an adult population assessed by accelerometry. Med Sci Sports Exerc 39: 1502-1508. doi:10.1249/mss.0b013e3180a76de5. PubMed: 17805081. 17805081

[46] Avesani CM, Trolonge S, Deléaval P, Baria F, Mafra D et al. (2012) Physical activity and energy expenditure in haemodialysis patients: an international survey. Nephrol Dial Transplant 27: 2430-2434. PubMed: 22172727. 22172727 10.1093/ndt/gfr692

[47] Bhasin SK, Dwivedi S, Dehghani A, Sharma R (2011) Conventional risk factors among newly diagnosed coronary heart disease patients in Delhi. World. J Cardiol 3: 201-206. 10.4330/wjc.v3.i6.201 PMC3139041 21772946

[48] Ma G, Luan D, Li Y, Liu A, Hu X et al. (2008) Physical activity level and its association with metabolic syndrome among an employed population in China. Obes Rev 9 Suppl 1: 113-118. doi:10.1111/j.1467-789X.2007.00451.x. PubMed: 18307712. 18307712

[49] Leon AS, Connett J, Jacobs DR Jr., Rauramaa R (1987) Leisure-time physical activity levels and risk of coronary heart disease and death. The Multiple Risk Factor Intervention Trial. JAMA 258: 2388-2395. doi:10.1001/jama.258.17.2388. PubMed: 3669210. 3669210

